# Correction to: CD16xCD33 Bispecific Killer Cell Engager (BiKE) as potential immunotherapeutic in pediatric patients with AML and biphenotypic ALL

**DOI:** 10.1007/s00262-021-03040-0

**Published:** 2021-09-07

**Authors:** Sarah B. Reusing, Dan A. Vallera, Angela R. Manser, Titus Watrin, Sanil Bhatia, Martin Felices, Jeffrey S. Miller, Markus Uhrberg, Florian Babor

**Affiliations:** 1 Institute for Transplantation Diagnostics and Cell Therapeutics, Heinrich Heine University, Düsseldorf, Germany; 2 Department of Pediatric Oncology, Hematology and Clinical Immunology, Centre for Child and Adolescent Health, Medical Faculty, Heinrich Heine University, Moorenstraße 5, 40225 Düsseldorf, Germany; 3 Department of Therapeutic Radiology-Radiation Oncology, Masonic Cancer Center, University of Minnesota, Minneapolis, MN USA; 4 Department of Medicine, Division of Hematology, Oncology and Transplantation, Minneapolis, MN USA

## Correction to: Cancer Immunology, Immunotherapy 10.1007/s00262-021-03008-0

The original version of this article unfortunately contained a mistake. Author name Titus Watrin was incorrectly written as Titus Vatrin.

